# Care Needs of Highly Complex Chronic Patients in the Canary Islands: An Observational Study

**DOI:** 10.3390/nursrep13010001

**Published:** 2022-12-20

**Authors:** Martín Rodríguez-Álvaro, Domingo Ángel Fernández-Gutiérrez, Antonio Cabeza-Mora, Rosario Barrios-Torres, Nursing Methodology Group General Directorate of Health Care Programs of the Canary Islands Health Service (Primary Care), Pedro Ruymán Brito-Brito

**Affiliations:** 1 The Canary Islands Health Service, Nursing Department, Faculty of Healthcare Sciences, University of La Laguna, 38200 Santa Cruz de Tenerife, Spain; 2 Primary Care Management of Gran Canaria, The Canary Islands Health Service, 35006 Las Palmas de Gran Canaria, Spain; 3 Nursing Methodology Group, General Directorate of Health Care Programs of the Canary Islands Health Service (Primary Care), The Canary Islands Health Service, 35006 Las Palmas de Gran Canaria, Spain

**Keywords:** chronic disease, primary health care, standardized nursing terminology, risk adjustment

## Abstract

In the last few decades, the impact of chronic health conditions on health systems, as well as on the quality of life, frailty, and dependence of those affected, has been brought to light. The objective of this study was to describe the population care needs of highly complex chronic patients (HCCPs). Methods: An epidemiological observational study was conducted. Results: A total of 13,262 patients were identified, 51% of which were elderly women. Among all patients, 84.4% had received a nursing assessment related to health patterns. Three diagnoses were established in 25% of the sample: readiness for enhanced health management, impaired skin integrity, and risk for falls. There were significant differences according to age, most importantly in terms of impaired skin integrity (39% of patients under 80 years old). Risk for falls, social isolation, situational low self-esteem, chronic low self-esteem, impaired home maintenance, anxiety, ineffective health management, ineffective coping, impaired memory, insomnia, and self-care deficits were more common in those living alone. A total of 37 diagnoses featured differences according to frailty/dependence. Approximately 23% of HCCPs suffered from frail elderly syndrome. Conclusions: This study presents the most common care needs of HCCPs, describing the sociodemographic profile of this part of the population. The planning of HCCP care varies in nature. Factors such as the dependence level and frailty of these people should be taken into consideration.

## 1. Introduction

In the last few decades, the impact of chronic health conditions on health systems, as well as on quality of life, frailty, and dependence of those affected, has been brought to light. Chronic diseases, such as heart failure, high blood pressure, hyperlipidemia, and diabetes, are long-term, slow-progressing health problems that have an impact on quality of life for both patients and their caregivers. Consequently, care models should be able to properly address the problems brought about by chronic conditions. Care models have been developed that are aimed at age and frailty, such as the Chronic Care Model (CCM) [1], the Program of Research to Integrate Services for Maintenance of Autonomy (PRISMA) [2], and Kaiser Permanente (KP) [3].

According to the 2017 Spanish National Health Survey (ENS), 22.8% of the population reports having a chronic limitation when carrying out their daily activities, with 4.3% having severe limitations [4]. In the Autonomous Community of the Canary Islands (ACCI), an estimated 6% of people over 16 years old are severely limited when carrying out their daily activities due to suffering from some kind of health condition [5]. 

The *Estrategia para el Abordaje de la Cronicidad* [Strategy for Tackling Chronicity] (EAC) of the Spanish National Health System (SNHS) highlights the need to stratify the population in order to predict the needs of those suffering from chronic conditions. This stratification is linked to a comprehensive assessment of patients’ medical, care, functional, and social needs [6]. 

There are multiple population groups for the stratification of chronic patients, e.g., adjusted morbidity groups (AMGs), clinical risk groups (CRGs) [7], and adjusted clinical groups (ACGs) [8]. A project to stratify the Spanish population was carried out by AMG in the SNHS, providing a multi-level tool, which complies with predefined profiles for each level according to a series of health and social care variables, and developing a risk prediction model [9]. 

The AMG is a stratification tool that has been proven useful and fit-for-purpose, just like the other existing groups [10]. This stratification follows the Kaiser pyramid model, which establishes percentiles on the basis of complexity levels [3]. In this way, we established four population complexity levels: (1) population without chronic conditions; (2) low-risk chronic population, i.e., people whose individual complexity value is lower than the 80th percentile of the chronic condition population; (3) moderate-risk chronic population, i.e., people whose individual complexity value is in the 80–95th percentile of the chronic condition population; (4) high-risk chronic population, i.e., people whose individual complexity value is higher than the 95th percentile of the chronic condition population.

As indicated in the EAC in the ACCI, highly complex chronic patients (HCCPs) make up around 5% of the population. These patients require a case management approach to care: personalized care according to their needs to optimize the coordination of system resources, improve the quality of life of patients and caregivers, and avoid emergency admissions and hospitalizations [6].

Primary care (PC) is at the core of complex chronic patient care. Community nurses can identify individual, family, environmental, and community care needs. Keeping in mind the changes that have taken place in the last few decades regarding population structure, with increasing degrees of frailty, dependence, and chronic conditions, nurse identification of care needs should contribute to better individual care for the HCCP. Furthermore, this should be taken into consideration when deciding health and resource management policies. On that note, in their assessment report and priority guidelines, the EAC once again highlighted the importance of community nurses in this type of patient care, underlining the need to develop the role of these professionals regarding individuals with chronic conditions, thereby promoting the application of comprehensive care assessments [11].

The current total population of the Autonomous Community of Canary Islands is 2.207 million people. PC professionals in the Canary Islands have maintained electronic health records (EHRs) since 2010. Here, they have access to HCCP stratification information, the AMG color (indicating complexity), and the diagnostic descriptive labels used for the over-14 age group. Additionally, the professional team regularly reviews all the available information and can, according to the criteria, actively include patients in the HCCP program using information from the AMG, and other clinical variables: the Barthel index, Pfeiffer’s test, polypharmacy, admissions, etc.

Community nurses have access to a specific module in the EHR in order to log the care they provide. This module follows nursing process logic [12]. The nurse creates a care plan by starting with an assessment based on Marjory Gordon’s health patterns (HPs) [13]. This assessment explores the individual’s functional status in depth using 11 HPs: health perception/health management; nutrition/metabolism; elimination; physical activity/exercise; sleep/rest; cognition/perception; self-perception/self-concept; role/relationships; sexuality/reproduction; coping/stress tolerance; values/beliefs. The assessment of each HP is with the help of complimentary measuring instruments such as scales, tests, and questionnaires. After completing the assessment, the professional should give their clinical judgement and record the functional status result of each HP as normal, altered, risk of alteration, or nonassessable.

In order to identify and diagnose individual care needs, as well as plan their care, nurses use the NANDA-I standardized taxonomy [14], the health outcomes section of the Nursing Outcomes Classification (NOC) [15], and the care interventions section of the Nursing Interventions Classification (NIC) [16]. In Spain, the use of standardized nursing languages (SNLs) is regulated by the Royal Decree 1093/2010 of 3 September and is part of the minimum dataset in clinical reports category of the SNHS [17].

The aim of this study is to use the NANDA-I classification system and the SNLs to describe the population care needs of HCCPs in the Canary Islands and analyze how they relate to other sociodemographic and clinical variables, such as frailty and dependence levels.

## 2. Materials and Methods

### 2.1. Design and Sampling Method

This was an observational, descriptive, cross-sectional, epidemiological study. The study population was HCCPs living in the Canary Islands, specifically those who (according to the AMG stratification system) are in the 99.5th complexity percentile or higher, or those who have been included by referral professionals (PC physician or nurse) in the HCCP management process. All ACCI patients meeting one of these two criteria on 1 January 2019 were included in the study. The study is reported according to STROBE reporting guidelines for observational research [18].

### 2.2. Study Setting

PC in the ACCI is split into seven areas and 112 basic healthcare districts (ZBSs) in which approximately two million users receive care. There is one EHR for the entire region, known as Drago-AP. It is a tool that works using an ORACLE database. As mentioned before, The Drago-AP EHR offers a specific module for recording and planning patient care [19] using a structured assessment by HP, leading to a determination of the care needs identified in the NANDA-I nursing diagnoses (NDs). The predicted outcome criteria register and the register of interventions or care to be carried out are based on the NOC and NIC classifications, which are internationally used SNLs.

### 2.3. Variables

The sociodemographic and clinical variables of the HCCPs were recorded in the Drago-AP EHR. Regarding NDs, the research team chose 60 NANDA-I statements for their study, as well as 40 medical diagnoses relating to chronic health problems. The team used the results of the assessment by HP to describe the functional status of the population. Comorbidity was determined using the Charlson comorbidity index (short version), a simple, validated, and readily acceptable method of determining the risk of mortality from comorbid disease. It has been used as a predictor of long-term survival and prognosis [20]. To describe the sociofamiliar situation, the results of the Gijón scale were used, made up of five items (family situation, economic situation, housing, relationships, and social support) [21]. The Pfeiffer’s test was used to assess cognitive impairment [22]. The level of dependence was determined with the help of the Barthel index, an instrument widely used to this end that measures the capacity of the person for the execution of 10 basic activities in daily life, obtaining a quantitative estimation of the subject’s level of dependency [23]. The variables concerning the use of healthcare resources were the number of visits per year to the nurse and the PC physician (general practitioner), as well as the care received at home from the community liaison nurse.

### 2.4. Data Collection Procedure

An anonymized database was created in an Excel file containing all the information needed to carry out the analysis in accordance with the study objectives. Each dataset was extracted from EHR employing an automated search program. Each EHR was coded alphanumerically, which respects the principles of confidentiality and protection of personal data. The database was exported in Excel format to the IBM SPSS© v.25.0 statistical program (IBM Corp. Released 2017. IBM SPSS Statistics, Version 25.0. Armonk, NY: IBM Corp) for cleaning and subsequent analysis.

Although this investigation was not an experimental design, nor did it feature any type of intervention, permission was requested and subsequently granted from the corresponding Research Ethics Committee.

### 2.5. Data Analysis

The description of nominal variables was performed by examining the frequency of the categories, and means (SDs) or medians (P5–P95) were used for the scale variables. To compare differences, the bivariate analysis was conducted using the chi-squared test, and Cramér’s *V* was employed to evaluate the intensity of the relationship. To assess the magnitude of Cramér’s coefficients, Rea and Parke’s criteria [24] were taken as reference: 0.00–0.10 = negligible; 0.10–0.20 = weak; 0.20–0.40 = moderate; 0.40–0.60 = relatively strong; 0.60–0.80 = strong; 0.80–1.00 = very strong. All tests were two-tailed and performed at an alpha significance level <0.05 using the IBM^®^ Statistical Package for the Social Sciences (SPSS) v.25.0.

## 3. Results

### 3.1. Sociodemographic and Clinical Profile of the Highly Complex Chronic Patient in the Canary Islands

A total of 13,262 HCCPs in or above the 99.5th percentile were identified. Of the sample, 51% (*n* = 6724) were elderly women (Table 1) with a mean age of 80 years old (SD = 10.5; median = 82; mode = 86). The most populated islands, Tenerife and Gran Canaria, housed 83% of Canary Island HCCPs: 48% (*n* = 6341) and 35% (*n* = 4596) respectively.

Furthermore, 33.8% of HCCPs were classed as such by PC professionals according to frailty/dependence variables. In this group, 46% (*n* = 1991) were identified as a dependent older person, 34% (*n* = 1497) were identified as frail, and 20% (*n* = 862) were identified as independent older persons. A total of 11% (*n* = 1440) of HCCPs lived alone. Within this group, 13.5% (*n* = 907) were women (*p* < 0.001; Cramér’s *V* = 0.086) and 12.7% (*n* = 1011) were people over the age of 80 (*p* < 0.001; Cramér’s *V* = 0.071). 

Patients had an annual mean of 13 visits to the PC nurse (NDs = 20; median = 10) and 12 to the general practitioner (NDs = 7.5; median = 11). Moreover, 97% of patients were seen at least once by their physician and 95% by the nurse, as well as 20% by the liaison nurse. The percentage of HCCPs assessed by the liaison nurse rose to 38% when dealing with dependent older people. An HCCP is considered a frequent attender when they are above the 75th percentile for the number of annual visits (15 visits to the physician or nurse) and a very frequent attender when they are above the 90th percentile (18 visits to the physician or nurse) (Table 2). There are no differences by gender. Around 20% of dependent HCCPs are very frequent attenders. Additionally, 16% of participants in the study were hospitalized at least once in the previous year, with 1% being admitted five or more times. 

### 3.2. Predominant Health Problems

Almost all of the study population was diagnosed with heart failure (HF) and high blood pressure (HBP). Three in four suffered from hyperlipidemia and diabetes mellitus, and more than half suffered from osteoarthritis, ischemic heart disease, and chronic renal failure (CRF) (Table 3). In 11.5% of HCCPs (*n* = 1519), associated comorbidities were determined using the Charlson comorbidity index, with 64% (*n* = 978) being classed as patients with a high level of comorbidity.

Dependence when carrying out activities of daily living was assessed using the Barthel index for two in every 10 patients (*n* = 2879). Of these, 34% presented severe or total dependence (*n* = 1455). The presence of severe or total dependence was higher among women (36%; *n* = 884; *p* < 0.001; Cramér’s *V* = 0.071). A total of 2.7% of patients with severe or total dependence were in a higher social risk category, and 12.3% suffered from a severe cognitive impairment. Cognitive screening was recorded using Pfeiffer’s test in 33.8% (*n* = 4489) of patients. Of these, 7% (*n* = 299) presented with severe cognitive impairment, 27% (*n* = 1196) presented with deficits to intellectual functioning, and 67% presented with normal cognitive and intellectual functioning. This screening was performed on six in 10 women and four in 10 men. The sociofamiliar situation was assessed using the Gijón scale in only 4.2% (*n* = 559) of HCCPs, with the majority (68.5%) being ranked as having intermediate social risk.

### 3.3. Assessment of Functional Status and NDs

Some 84.4% of HCCPs had a nursing assessment record through the HPs. In 32% (*n* = 4261) of cases, an assessment of all HPs was recorded. The functional status results are detailed in Table 4. The HP for which information was most frequently recorded was perception/health management (74.2%), while the least frequently recorded was values/beliefs (35.7%). A total of 23% of participants presented with problems related to physical activity/exercise, along with 22% related to urinary function, 21% related to self-care, 13% related to sleep/rest, and 5% related to self-esteem. In 47% of HCCPs (*n* = 6278), at least one of the study NDs was recorded. Some 34% of participants (*n* = 4526) presented between one and 10 NDs, 12% (*n* = 1640) presented between 11 and 20 NDs, and 1% (*n* = 112) presented more than 20 NDs. The following three NDs were identified for one in every four patients: readiness for enhanced health management, impaired skin integrity, and risk for falls.

Table 5 shows all of the NDs and their significance by gender. In 43% of NDs, there were significant differences by gender, with each of them being more common in women. The NDs chronic pain, stress urinary incontinence, and anxiety showed the best association values as per Cramér’s *V* values, although these values were weak. None of the associations between gender and ND presented as moderate or relatively strong. 

The relationship NDs and associations by age (80+ years old), living alone, and dependence are described in Table 6. A group of NDs presented significant differences depending on the age range, e.g., impaired skin integrity was present in 39% of those under 80 (Cramér’s *V* = 0.105). Problems such as risk for falls, social isolation, situational low self-esteem, chronic low self-esteem, impaired home maintenance, anxiety, ineffective health management, ineffective coping, impaired memory, insomnia, and self-care deficits (bathing, dressing, and feeding) were significantly more common among HCCPs who lived alone. A group of 37 NDs showed significant differences (*p* < 0.005) depending on if the patient was independent, frail, or dependent. None of them showed a moderate or relatively strong strength of association. Self-care deficits (bathing, dressing, feeding, and toileting) and risk for falls showed a weak association. A total of 8.7% of the study population was diagnosed with frail elderly syndrome. However, there was a proportion of patients (16%, *n* = 1942) who, despite not receiving these NDs, met two or more of the defining characteristics (DCs) of the ND. The prevalence of these DCs/NDs was as follows: bathing self-care deficit (81%), dressing self-care deficit (70%), impaired walking (61%), impaired physical mobility (51.5%), feeding self-care deficit (45%), toileting self-care deficit (44%), impaired memory (32%), social isolation (9%), and hopelessness (4%).

In combining these two groups, 23% (*n* = 3100) of HCCPs did indeed suffer from frail elderly syndrome, with six in 10 patients from this subgroup being women. It is noteworthy that, in 91% of cases (*n* = 2818) meeting these characteristics, there were records of assessment by HPs.

## 4. Discussion

The most prevalent characteristics of the HCCP are well identified in the literature. Within these characteristics is the presence of comorbidities, increased use of emergency services, several hospitalizations per year, loss of personal independence, polypharmacy, and the presence of certain illnesses such as HF or COPD [25]. The classification of patients using the AMG is useful in planning care, but it is necessary to continue improving the quality of available information in the EHR so that it can be used in the stratification system. This is demonstrated by the large variability in healthcare costs for users in similar complexity percentiles [26]. In this respect, the weight of care needs identified through NDs has not been sufficiently researched or assessed for these types of patients.

Nurse identification of care needs contributes to the improvement of personalized care of the HCCP can and should help inform health policies and manage resources in epidemiological nursing. NDs can be used as key descriptors of population care needs for the profile under study, especially in more complex cases. A systematic exercise of epidemiological nursing allows for the creation of sentinel networks for care needs [27]. Furthermore, the inclusion of these needs in the EHR and in the stratification algorithms can improve predictions about degrees of complexity [28], as well as elevate the explanatory power of the use of healthcare resources [29].

In our context (PC in the Canary Islands within the public SNHS), several studies have addressed and discussed the care needs identified for specific groups of patients. This is especially true in relation to psychosocial issues such as loss and mourning [30,31], for frail and dependent patients in particular, with characteristics similar to the population in this study [32].

It is widely known that nurses often record fewer activities than what they actually perform [33]. Traditionally, nursing documentation has consisted of narrative notes that are often long, ambiguous, and redundant [34]. In terms of the use of NDs in the EHR, the tendency of nurses to only record diagnostic labels has been highlighted, making the total of said records very high in number [35]. Therefore, with regard to the functional status assessment using HPs, it should be highlighted that information is recorded in more than 84% of HCCPs. To put these data into context, according to a report released in July 2022 on the use of the EHR in the Canary Islands, only 27% of the adult population had an HP assessment in their records. As such, for HCCPs, nurses record a great deal more information in the area of standardized HPs. This study’s results are consistent with previously reported studies, which identified the most assessed HP is health perception/health management [36]. 

Three diagnostic labels were identified in one in every four patients: readiness for enhanced health management, impaired skin integrity, and risk for falls. The identification of these problems should help with managing HCCP care.

The high prevalence of the first of these diagnoses, corresponding to the domain of health promotion and self-management, i.e., readiness for enhanced health self-management, is common in the EHR. As stated in its definition, in the most recent NANDA-I classification of NDs, this label is used to indicate that the person has ‘a pattern of satisfactory management of symptoms, treatment regimen, physical, psychosocial, and spiritual consequences with lifestyle changes inherent in living with a chronic condition, which can be strengthened’ [15]. Women, under-80s, and nondependent persons present this ND very frequently, giving the patient an active role in the planning and provision of their care.

There are no previous studies that estimated the prevalence of impaired skin integrity in the HCCP within our context. However, the most appropriate comparison framework for this study’s findings could be the study on the prevalence of pressure injuries and other skin lesions related to dependence in Spanish PC facilities, performed in 2017 [37]. This investigation, led by the Spanish National Advisory Group for the Study of Pressure Ulcers and Chronic Wounds (GNEAUPP), found that the prevalence of pressure injuries and other skin lesions related to the dependence of over-65 s was 0.3%, while, in people who are part of home care programs, it was 6%. These results are considerably lower than the results of our study. In our investigation, prevalence varied on the basis of whether the person was independent (41%), frail (38%), or dependent (26%). However, it was found that, with less dependence, there was more impaired skin integrity. This discrepancy may arise from the fact that, firstly, the samples were not directly comparable. Secondly, this is an ND with a high abstraction level, meaning that it is too broad to carry out a precise interpretation. Any abnormality of the epidermis or dermis will come under this category. The aim of our study was not to discuss how much detail an ND should have; however, it is clear that, in this specific case, both in clinical practice and in interpreting the data, it should at least be accompanied by its DCs, i.e., its clinical manifestations, as well as the related factors.

In terms of risk for falls, it seems to be an underestimation that only one in four HCCPs suffer from this potential issue. The literature shows that three in 10 people over the age of 65 living in the community suffer falls each year [38]. In this study population, in seven out of 10 patients assessed, the nurse indicated that the HP physical activity/exercise was altered. People with higher levels of disability present a greater risk [39], which agrees with the data from our investigation, where the prevalence of risk for falls in dependent, frail, and independent people was 46%, 38%, and 16%, respectively. In future studies, it would be worthwhile to include an assessment of intrinsic and extrinsic factors that increase risk, such as those identified by other authors as more frequent factors. These include visual impairment, mobility issues, history of falls, inadequate resources in the bathroom, and loose rugs [40]. In addition, a validated scale, test, or tool to measure risk should be considered [41].

Logically, at present, it seems that the ND that most fits the HCCP profile may be frail elderly syndrome. It was included in the NANDA-I classification in 2013 and is defined as the dynamic state of unstable equilibrium that affects the older individual experiencing deterioration in one or more domains of health (physical, functional, psychological, or social) and leads to increased susceptibility to adverse health effects, particularly disability. It has a level of evidence of 2.1, meaning that, until now, it has not been clinically validated with a large sample of patients. According to NANDA-I, a syndrome is defined as ‘a clinical judgement concerning a specific cluster of nursing diagnoses that occur simultaneously and are best addressed together and through similar intervention’ [42], and it must include a minimum of two NDs, meaning it has a significant impact on several human responses [43]. 

Frail elderly syndrome is directly present in only 9% of HCCPs. However, when we add patients who meet diagnostic criteria to this group, we found that 23% of patients suffered from this issue, which would position it as the fourth most common ND. The infrequent recording of this syndrome by community nurses in PC in our context, compared to the number of patients who meet the diagnosis criteria, could be due to various reasons. On the one hand, it was included in the NANDA-I classification of 2013, which makes it a more recent addition compared to other NDs that have been part of the classification system for longer. On the other hand, it seems to be easier to identify the records of each ND that makes up the syndrome rather than the syndrome itself. To properly plan and manage care, the assessment should be more in depth and specify which DC led to the diagnosis. 

The majority of patients diagnosed with frail elderly syndrome or who met two or more of the DCs for this issue were women, at 60%. This coincides with data from the ENS in Spain, in which women reported a higher frequency of limitations than men. This gap by gender is common across all disability indicators [4]. 

In Spain, 20–40% of older people experience social isolation and loneliness [44]. This figure is considerably higher than what we found in our study on HCCPs, where 11% of patients live alone and only 2% of them present the NDs of social isolation and risk for loneliness, according to EHR records. The consequences of loneliness on health and quality of life for older people are well known [45], being linked to a considerably higher morbidity rate [46,47], which agrees with the results of our investigation. Other problems referred to previously are more common in HCCPs who live alone: risk for falls, social isolation, situational low self-esteem, chronic low self-esteem, impaired home maintenance, anxiety, ineffective health management, ineffective coping, impaired memory, insomnia, and self-care deficits (bathing, dressing, and feeding). This determines the type of care needs of these HCCPs who live alone and informs their care plan.

It is noteworthy that more than 95% of patients included in the investigation were seen by their nurse or general practitioner in the last year. As a result, these patients are well known by the referring healthcare team, making their assessment, management, and care much easier. Among other factors, advanced age and chronicity are associated with frequent attendance and overuse of PC [48]. It is known that older patients, higher-risk patients, and higher-complexity patients use health resources more frequently [49]. There is no consensus in the literature to describe a hyperfrequent-attending patient, as it generally uses arbitrary methods instead [50]. With the variables under study, we were unable to determine the profile of a hyperfrequent-attending HCCP or the needs left uncovered that may have arisen from this increased demand. 

In theory, the liaison nurse or nurse case manager is the health professional who should lead HCCP care. As suggested by Mármol and López, independent of the conceptual framework, all of the implemented strategies and initiatives point to the PC, particularly the community nurse as guarantors of providing care to chronically ill patients, their families, and the community [51]. Theoretically, follow-up by the liaison community nurse has a positive impact on coverage and hospitalization outcomes, pressure injuries, falls, caregiver role strain, assessing social risk, and home visits from referral healthcare professionals in PC. Therefore, this could be an efficient alternative in providing care to the polypathological, polymedicated, and dependent groups of the population [52]. However, similar to findings from our study, only 20% of these patients have been visited by the liaison nurse. This could be related to the unequal implementation of this figure in the ACCI, whereby these professionals cannot cover every basic healthcare district; rather, they are distributed, in general, in areas where the population is more spread out and hard to access, primarily due to the relief of the terrain.

This investigation presented some limitations for consideration, particularly those deriving from its retrospective nature, i.e., carried out using EHR records and not with a direct longitudinal assessment of each chronic patient. In addition, in some cases, the diagnostic label could be interpreted with a high level of abstraction, being too broad. It may be worthwhile in future studies to explore other components of NDs, such as DCs, related factors, and risk factors within the initial diagnostic labels identified by nurses. Furthermore, regarding measuring instruments used (scales, tests, or questionnaires), each one of their dimensions should be examined with, instead of just, their final score. This would allow for the identification of specific areas of dysfunction regarding the components of said instruments. Within this line of research, for the identification of the HCCP, it is suggested to include certain NDs considered as especially sensitive among these patients, such as risk for falls, bathing self-care deficit, impaired skin integrity, and frail elderly syndrome. Departing from this epidemiological study, a new working hypothesis can be formulated and new research plans can be built that allow determining the weight and influence of care needs identified by the community nurse in terms of the degree of complexity of the chronic patient.

## 5. Conclusions

This study presented the most frequent care needs (NDs) of HCCPs in an autonomous community of the SNHS, the Canary Islands, describing the sociodemographic profile of this part of the population. Planning of HCCP care is varied in nature. Healthcare components, such as level of dependence and frailty of these patients should be taken into consideration, among other factors. The most common care needs of these types of patients are the disposition for better health management, the deterioration of skin integrity, and the risk of falls. Factors such as the level of dependency and fragility of these people must be taken into account when planning the care of health professionals.

In any case, future studies must address the importance and repercussions of these needs, recorded in the EHR, to be able to know their exact predictive capacity regarding HCCP health complications, as well as their possible inclusion as complementary factors of interest to improve healthcare and health outcomes.

## Figures and Tables

**Table 1:** Study population distribution by age group.

	Total		Male		Female	
	*n*	%	*n*	%	*n*	%
Young adult (19–24)	2	0.0	1	0.0	1	0.0
Adult (25–44)	66	0.5	27	0.4	39	0.6
Middle aged (45–64)	959	7.2	517	7.9	442	6.6
Elderly (65–79)	4253	32.1	2417	37.0	1836	27.3
Elderly (80+)	7982	60.2	3576	54.7	4406	65.5

**Table 2:** Distribution of chronic patients with high-frequency attendance separated by gender, dependence, and visits with community liaison nurse.

	Hyperfrequent Attender						Extremely Frequent Attender					
	Nurse			Physician			Nurse			Physician		
	%	*Cv*	*p*	%	*Cv*	*p*	%	*Cv*	*p*	%	*Cv*	*p*
Male	49.0	0.01	0.9	44.6	0.05	<0.001	50.5	0.08	0.356	41.5	0.04	<0.001
Female	51.0			55.4			49.5			58.5		
Dependent older person	20.4	0.08	<0.001	18.7	0.06	<0.001	22.7	0.07	<0.001	18.8	0.03	0.001
Visited at least once by CLN	28.3	0.12	<0.001	25.6	0.09	<0.001	34.1	0.13	<0.001	33.5	0.10	<0.001

Note: CLN—community liason nurse. Strength of association criteria based on value of Cramér’s *V*: negligible = from 0.00 to <0.10; weak = from 0.10 to <0.20; moderate = from 0.20 to <0.40; relatively strong = from 0.40 to <0.60; strong = from 0.60 to <0.80; very strong = from 0.80 to 1.00. A highly complex chronic patient is considered a hyperfrequent attender (HF) when they are above the 75th percentile for number of annual visits (15 visits to the physician or nurse) and an extremely frequent attender (EF) when they are above the 90th percentile (18 visits to the physician or nurse).

**Table 3:** Prevalence of medical diagnoses under study.

Medical Diagnoses	*n*	%
HF	13,247	99.9
HBP	12,786	96.4
Hyperlipidemia	10,178	76.7
Diabetes	10,154	76.6
Osteoarthritis	9380	70.7
IHD	7229	54.5
CRF	7125	53.7
Dysrhythmia	6485	48.9
Obesity	6232	47.0
Depression	6173	46.5
COPD	5947	44.8
Anaemia	4576	34.5
Cardiac conduction disorder	4508	34.0
CVA	4246	32.0
Valvular heart disease	4088	30.8
Asthma	3834	28.9
Osteoporosis	3811	28.7
Arthritis	3756	28.3
Urinary tract infection	3405	25.7
Anxiety	3370	25.4
Thyroid disorder	3367	25.4
Dementia	3256	24.6
Prior neoplasm	2598	19.6
Cardiopulmonary disease	1609	12.1
Cardiomyopathy	1485	11.2
Active neoplasm	1485	11.2
Glaucoma	1479	11.2
Alcohol use disorders	1446	10.9
Pneumonia	1380	10.4
Paralysis	930	7.0
Parkinson’s	791	6.0
Respiratory failure	767	5.8
Aortic aneurysm	752	5.7
Gastrointestinal bleeding	695	5.2
Epilepsy	611	4.6
Metastasis	603	4.5
Liver disease	597	4.5
Septicemia	466	3.5
Schizophrenia	426	3.2
Non-Hodgkin’s lymphoma	294	2.2
Lupus	117	0.9

Note: HF, heart failure; HBP, high blood pressure; IHD, ischemic heart disease; CRF, chronic renal failure; COPD, chronic obstructive pulmonary disease; CVA, cerebrovascular accident.

**Table 4:** Functional status of the population according to the results of the assessment by HPs.

	Non-Assessable		Normal		Risk of Alteration		Altered	
	*n*	%	*n*	%	*n*	%	*n*	%
Health perception/health management	111	1.1	2426	24.6	2063	21.0	5244	53.3
Nutrition/metabolism	20	0.2	2272	24.5	1791	19.3	5193	56.0
Elimination	16	0.2	2678	33.8	818	10.3	4409	55.7
Physical activity/exercise	28	0.3	1599	18.2	1176	13.4	5966	68.0
Sleep/rest	35	0.5	3576	49.6	947	13.1	2646	36.7
Cognition-perception	79	1.1	1860	25.0	954	12.8	4545	61.1
Self-perception/self-concept	607	10.6	2138	37.2	953	16.6	2051	35.7
Role/relationships	78	1.3	3385	54.9	1223	19.8	1485	24.1
Sexuality/reproduction	1654	33.0	3035	60.5	106	2.1	218	4.3
Coping/stress tolerance	523	9.9	2727	51.5	945	17.8	1101	20.8
Values/beliefs	1345	28.4	2844	60.0	264	5.6	284	6.0

**Table 5:** Nursing diagnoses under study, with significance by gender.

Nursing Diagnoses	Total				Male		Female	
	*n*	%	*Cv*	*p*	*n*	%	*n*	%
Readiness for enhanced health self-management	4464	33.7	0.01	<0.001	2113	32.3	2351	35
Impaired skin integrity	4303	32.4	0.01	0.512	2139	32.7	2164	32.2
Risk for falls	3279	24.7	0.10	<0.001	1323	20.2	1956	29.1
Bathing self-care deficit	2377	17.9	0.09	<0.001	947	14.5	1430	21.3
Risk for unstable blood glucose level	2296	17.3	0.02	<0.001	1089	16.7	1207	18
Impaired walking	2017	15.2	0.09	<0.001	788	12.1	1229	18.3
Dressing self-care deficit	2005	15.1	0.08	<0.001	795	12.2	1210	18
Chronic pain	1933	14.6	0.13	<0.001	656	10.0	1277	19.0
Ineffective breathing pattern	1896	14.3	0.02	<0.001	878	13.4	1018	15.1
Impaired urinary elimination	1878	14.2	0.09	<0.001	726	11.1	1152	17.1
Impaired comfort	1678	12.7	0.06	<0.001	706	10.8	972	14.5
Impaired physical mobility	1661	12.5	0.07	<0.001	662	10.1	999	14.9
Risk for impaired skin integrity	1520	11.5	0.04	<0.001	669	10.2	851	12.7
Ineffective health management	1469	11.1	0.03	<0.001	661	10.1	808	12.0
Feeding self-care deficit	1385	10.4	0.07	<0.001	552	8.4	833	12.4
Insomnia	1339	10.1	0.08	<0.001	493	7.5	846	12.6
Toileting self-care deficit	1289	9.7	0.08	<0.001	486	7.4	803	11.9
Frail elderly syndrome	1158	8.7	0.06	<0.001	458	7.0	700	10.4
Anxiety	1154	8.7	0.10	<0.001	376	5.8	778	11.6
Impaired home maintenance	1122	8.5	0.09	<0.001	380	5.8	742	11
Impaired memory	1068	8.1	0.08	<0.001	390	6.0	678	10.1
Functional urinary incontinence	910	6.9	0.09	<0.001	301	4.6	609	9.1
Decreased diversional activity engagement	857	6.5	0.06	<0.001	333	5.1	524	7.8
Activity intolerance	682	5.1	0.05	<0.001	263	4.0	419	6.2
Chronic sorrow	654	4.9	0.08	<0.001	211	3.2	443	6.6
Readiness for enhanced self-care	541	4.1	0.01	0.265	254	3.9	287	4.3
Ineffective coping	501	3.8	0.04	<0.001	201	3.1	300	4.5
Chronic confusion	468	3.5	0.06	<0.001	162	2.5	306	4.6
Urge urinary incontinence	433	3.3	0.06	<0.001	139	2.1	294	4.4
Situational low self-esteem	416	3.1	0.02	0.005	177	2.7	239	3.6
Impaired gas exchange	413	3.1	0.00	0.645	199	3.0	214	3.2
Risk for activity intolerance	404	3.0	0.02	0.019	176	2.7	228	3.4
Impaired transfer ability	308	2.3	0.02	0.012	130	2.0	178	2.6
Risk for frail elderly syndrome	299	2.3	0.02	0.042	130	2.0	169	2.5
Social isolation	298	2.2	0.03	0.002	121	1.9	177	2.6
Risk for loneliness	266	2.0	0.04	<0.001	99	1.5	167	2.5
Impaired swallowing	257	1.9	0.03	<0.001	99	1.5	158	2.3
Impaired verbal communication	256	1.9	0.00	0.781	124	1.9	132	2.0
Risk for disuse syndrome	238	1.8	0.02	0.045	102	1.6	136	2.0
Sleep deprivation	233	1.8	0.03	0.001	89	1.4	144	2.1
Disturbed sleep pattern	224	1.7	0.03	0.004	89	1.4	135	2.0
Chronic low self-esteem	220	1.7	0.05	<0.001	70	1.1	150	2.2
Stress urinary incontinence	215	1.6	0.11	<0.001	14	0.2	201	3.0
Impaired bed mobility	209	1.6	0.02	0.036	88	1.3	121	1.8
Acute confusion	161	1.2	0.01	0.184	71	1.1	90	1.3
Impaired wheelchair mobility	137	1.0	0.01	0.104	77	1.2	60	0.9
Hopelessness	135	1.0	0.03	<0.001	44	0.7	91	1.4
Risk for situational low self-esteem	134	1.0	0.02	0.015	52	0.8	82	1.2
Powerlessness	117	0.9	0.01	0.385	53	0.8	64	1.0
Reflex urinary incontinence	97	0.7	0.01	0.164	41	0.6	56	0.8
Risk for acute confusion	87	0.7	0.01	0.403	39	0.6	48	0.7
Risk for urge urinary incontinence	69	0.5	0.01	0.226	29	0.4	40	0.6
Defensive coping	62	0.5	0.01	0.364	27	0.4	35	0.5
Urinary retention	47	0.4	0.03	0.002	34	0.5	13	0.2
Self-neglect	46	0.3	0.01	0.326	26	0.4	20	0.3
Overflow urinary incontinence	30	0.2	0.02	0.080	10	0.2	20	0.3
Impaired standing	25	0.2	0.01	0.596	11	0.2	14	0.2
Chronic pain syndrome	21	0.2	0.01	0.143	7	0.1	14	0.2
Total urinary incontinence	14	0.1	0.02	0.037	3	0.0	11	0.2
Risk for chronic low self-esteem	8	0.1	0.00	0.169b	2	0.0	6	0.1
Impaired sitting	0	0.0	-	-	0	0.0	0	0.0

Strength of association criteria according to Cramér’s *V* values: 0.00–0.10 = negligible; 0.10–0.20 = weak; 0.20–0.40 = moderate; 0.40–0.60 = relatively strong; 0.60–0.80 = strong; 0.80–1.00 = very strong.

**Table 6:** Diagnostic labels under study, with significance by age group, living alone, and dependence.

Nursing Diagnoses	>80 Y.O.			Lives Alone			Dependent		
	*Cv*	*p*	%	*Cv*	*p*	%	*Cv*	*p*	%
Risk for falls	0.076	<0.001	27.4	0.055	<0.001	31.5	0.067	<0.001	31.6
Frail elderly syndrome	0.077	<0.001	10.5	0.026	0.03	10.8%	0.031	<0.001	10.8%
Risk for frail elderly syndrome	0.035	<0.001	2.7	0.026	0.073	2.9%	0.017	0.052	1.7%
Impaired sitting	-	-	0.0	-	-	0.0	-	-	0.0%
Impaired standing	0.018	0.043	0.3%	0.013	0.141b	0.3%	0.013	0.123b	0.1%
Self-neglect	0.036	<0.001	0.2%	0.029	0.001b	0.8%	0.007	0.431	0.3%
Readiness for enhanced self-care	0.023	0.008	3.7%	0.005	0.597	3.8%	0.034	<0.001	2.5%
Impaired bed mobility	0.021	0.014	1.8%	0.015	0.085	1.0%	0.072	<0.001	3.7%
Impaired home maintenance	0.039	<0.001	9.3%	0.048	<0.001	12.3%	0.044	<0.001	11.4%
Feeding self-care deficit	0.062	<0.001	12.0%	0.019	0.031	12.1%	0.115	<0.001	18.8%
Bathing self-care deficit	0.072	<0.001	20.2%	0.030	<0.001	21.3%	0.122	<0.001	29.0%
Dressing self-care deficit	0.070	<0.001	17.2%	0.019	0.027	17.1%	0.136	<0.001	26.7%
Toileting self-care deficit	0.059	<0.001	11.1%	0.004	0.635	10.1%	0.119	<0.001	18.1%
Acute confusion	0.006	0.51	1.3%	0.010	0.249	1.5%	0.021	0.016	1.8%
Chronic confusion	0.070	<0.001	4.6%	0.004	0.630	3.8%	0.084	<0.001	7.2%
Risk for acute confusion	0.007	0.42	0.7%	0.008	0.377	0.8%	0.008	0.357	0.5%
Impaired memory	0.710	<0.001	9.6%	0.037	<0.001	11.0%	0.073	<0.001	12.8%
Chronic sorrow	0.000	0.98	4.9%	0.28	0.01	6.7%	0.030	0.001	6.5%
Social isolation	0.011	0.22	2.1%	0.066	<0.001	5.1%	0.012	0.175	2.7%
Risk for loneliness	0.022	0.012	2.3%	0.140	<0.001	7.6%	0.004	0.611	1.9%
Chronic low self-esteem	0.029	0.001	1.4%	0.019	0.027	2.4%	0.015	0.088	2.1%
Risk for chronic low self-esteem	0.001	0.894b	0.1%	0.011	0.198bc	0.1%	0.010	0.234b	0.0%
Situational low self-esteem	0.035	<0.001	2.6%	0.025	4	4.4%	0.012	0.183	3.6%
Risk for situational low self-esteem	0.016	0.06	0.9%	0.011	0.214	1.3%	0.006	0.483	1.2%
Anxiety	0.074	<0.001	7.0%	0.036	<0.001	11.6%	0.017	0.045	7.5%
Impaired urinary elimination	0.010	0.23	14.5%	0.015	0.091	15.6%	0.024	0.005	16.2%
Stress urinary incontinence	0.010	0.23	1.7%	0.037	<0.001	2.9%	0.015	0.093	2.1%
Reflex urinary incontinence	0.001	0.90	0.7%	0.002	0.862	0.7%	0.056	<0.001	1.9%
Urge urinary incontinence	0.044	<0.001	3.9%	0.015	0.085	4.0%	0.008	0.339	3.6%
Functional urinary incontinence	0.077	<0.001	8.4%	0.025	0.004	8.7%	0.091	<0.001	12.3%
Total urinary incontinence	0.003	0.75	0.1%	0.004	0.680b	0.1%	0.006	0.501b	0.2%
Risk for urge urinary incontinence	0.031	<0.001	0.7%	0.008	0.331	0.7%	0.001	0.903	0.5%
Urinary retention	0.009	0.33	0.3%	0.020	0.021	0.7%	0.003	0.699	0.4%
Overflow urinary incontinence	0.003	0.69	0.2%	0.009	0.306b	0.3%	0.002	0.800b	0.3%
Ineffective health management	0.046	<0.001	9.9%	0.029	0.001	13.7%	0.020	0.022	9.6%
Impaired physical mobility	0.021	0.015	13.1%	0.018	0.038	14.2%	0.079	<0.001	18.7%
Impaired walking	0.062	<0.001	17.0%	0.030	0.001	18.3%	0.068	<0.001	21.0%
Impaired wheelchair mobility	0.019	0.029	0.9%	0.002	0.809	1.0%	0.055	<0.001	2.4%
Impaired transfer ability	0.024	0.005	2.6%	0.002	0.789	2.2%	0.047	<0.001	4.0%
Risk for disuse syndrome	0.019	0.025	2.0%	0.011	0.219	1.4%	0.029	0.001	2.7%
Impaired verbal communication	0.014	0.10	2.1%	0.014	0.114	1.4%	0.036	<0.001	3.1%
Ineffective coping	0.030	<0.001	3.3%	0.057	<0.001	6.9%	0.023	0.008	4.8%
Impaired skin integrity	0.105	<0.001	28.4%	0.010	0.252	31.1%	0.061	<0.001	25.6%
Risk for impaired skin integrity	0.031	<0.001	12.3%	0.003	0.729	11.7%	0.067	<0.001	16.6%
Risk for unstable blood glucose level	0.076	<0.001	15.0%	0.021	0.016	19.6%	0.031	<0.001	14.5%
Impaired gas exchange	0.014	0.11	2.9%	0.007	0.436	2.8%	0.016	0.069	2.5%
Ineffective breathing pattern	0.027	0.002	13.5%	0.010	0.269	13.3%	0.061	<0.001	9.2%
Defensive coping	0.007	0.39	0.4%	0.012	0.181	0.7%	0.007	0.411	0.4%
Activity intolerance	0.018	0.033	5.5%	0.024	0.006	6.7%	0.035	<0.001	7.0%
Risk for activity intolerance	0.010	0.26	3.2%	0.033	<0.001	4.7%	0.030	<0.001	1.8%
Decreased diversional activity engagement	0.017	0.050	6.8%	0.026	0.003	8.3%	0.035	<0.001	8.5%
Insomnia	0.010	0.27	9.9%	0.044	<0.001	13.9%	0.023	0.008	11.8%
Sleep deprivation	0.003	0.71	1.8%	0.020	0.023	2.5%	0.010	0.265	2.1%
Disturbed sleep pattern	0.001	0.91	1.7%	0.016	0.060	2.3%	0.001	0.906	1.7%
Impaired swallowing	0.027	0.002	2.2%	0.002	0.825	2.0%	0.065	<0.001	4.1%
Hopelessness	0.016	0.07	0.9%	0.015	0.078	1.5%	0.014	0.103	1.4%
Powerlessness	0.016	0.07	0.8%	0.016	0.060	1.3%	0.026	0.003	1.5%
Chronic pain	0.022	0.013	15.2%	0.054	<0.001	20.0%	0.031	<0.001	17.2%
Chronic pain syndrome	0.006	0.46	0.1%	0.010	0.227b	0.3%	0.011	0.188b	0.1%
Impaired comfort	0.530	<0.001	11.2%	0.004	0.687	13.0%	0.091	<0.001	5.4%

The chi-squared test was significant at the 0.05 level. Strength of association criteria according to Cramér’s *V* values: 0.00–0.10 = negligible; 0.10–0.20 = weak; 0.20–0.40 = moderate; 0.40–0.60 = relatively strong; 0.60–0.80 = strong; 0.80–1.00 = very strong. b More than 20% of the cells in this sub-table were predicted to be less than five. The results of the chi-squared test may not be valid. c The minimum predicted number for cells in this sub-table was less than one. The results of the chi-squared test may not be valid.

## Data Availability

The data presented in this study are available on request from the corresponding author.
